# Therapeutic Effects of Biobran, Modified Arabinoxylan Rice Bran, in Improving Symptoms of Diarrhea Predominant or Mixed Type Irritable Bowel Syndrome: A Pilot, Randomized Controlled Study

**DOI:** 10.1155/2014/828137

**Published:** 2014-08-05

**Authors:** Takeshi Kamiya, Michiko Shikano, Mamoru Tanaka, Keiji Ozeki, Masahide Ebi, Takahito Katano, Shingo Hamano, Hirotaka Nishiwaki, Hironobu Tsukamoto, Tsutomu Mizoshita, Yoshinori Mori, Eiji Kubota, Satoshi Tanida, Hiromi Kataoka, Noriaki Okuda, Takashi Joh

**Affiliations:** ^1^Department of Gastroenterology and Metabolism, Nagoya City University Graduate School of Medical Sciences, 1 Kawasumi, Mizuho-cho, Mizuho-ku, Nagoya 457-0036, Japan; ^2^Okuda Naika Clinic, 2-9-3 Hinata-cho, Mizuho-ku, Nagoya 467-0047, Japan

## Abstract

*Background*. Recently, it was revealed that low grade mucosal inflammation and/or immune imbalance of the lower digestive tract is one of the mechanisms involved in symptom generation in patients with irritable bowel syndrome (IBS). Biobran, arabinoxylan compound derived from rice bran, has been reported to have several biological actions such as anti-inflammatory and immune modulatory effects. So we investigated the therapeutic effects of Biobran in patients with IBS. *Method*. Forty patients with diarrhea predominant or mixed type IBS were randomly assigned to either a Biobran group for treatment with Biobran or a placebo group. Therapeutic efficacy and IBS symptoms were assessed subjectively by the patients after 4 weeks of administration. *Results*. The global assessment was effective in 63.2% of the Biobran group and in 30% of the placebo group (*P* < 0.05, Biobran group versus placebo group). Biobran group showed a significant decrease in the score of diarrhea and constipation and in CRP value. However, no significant changes were observed in the placebo group. *Conclusion*. The administration of Biobran improved IBS symptoms. It is likely that anti-inflammatory and/or immune modulatory effects of Biobran might be useful in IBS patients.

## 1. Introduction

Irritable bowel syndrome (IBS) is a common functional bowel disorder [1] in which abnormal discomfort or pain is associated with defecation or a change in bowel habit and with features of disordered defecation. Many studies [2–8] in Western countries and Japan have estimated the prevalence of IBS to be between 10% and 30% in the adult population. Furthermore, IBS is a chronic problem that affects all aspects of daily life and has a significant negative impact on quality of life (QOL). It is widely accepted that various factors contribute to the development of IBS symptoms. Although disturbed gastrointestinal motility, sensory hypersensitivity, and psychosomatic factors have been proposed as the possible reasons behind IBS [9], no final mechanisms have been agreed upon to date. Many IBS treatments are currently available, ranging from specifically designed drugs such as 5-HT_3_ antagonist and antidepressants to nonpharmacological therapies including hypnotherapy. Most of them are unsatisfactory, and new approaches to find the underlying pathogenesis are desirable.

Recently, there has been a general agreement that low grade mucosal inflammation and/or immune imbalance of the lower digestive tract are one of the mechanisms involved in symptom generation in IBS patients. Several studies [10–14] have reported inflammation in mucosal biopsies of the colon, rectum, and terminal ileum in IBS patients. These studies have shown that IBS patients have an increased number of inflammatory cells, including lymphocytes, dendritic cells, and mast cells in their mucosa, and 1/2 of IBS patients have microscopic inflammation compatible with microscopic colitis. Furthermore, IBS may occur in about 7%~30% of patients recovering from acute enterocolitis, a condition called postinfective IBS (PI-IBS) [15–17].

Modified arabinoxylan rice bran (Biobran) is highly water-soluble modified rice bran, composed of polysaccharides, mainly arabinoxylan hemicelluloses. It has been sold as a functional food for more than 10 years in over 40 countries, including some in North America, Europe, and Japan. Biobran has shown a range of immune modulatory activities. Some studies have reported that oral Biobran intake enhances natural killer (NK) cell activity in healthy humans and aged mice [18, 19] and the proliferation of lymphocytes (T and B cells) [20] and induces a significant increase in some of cytokines, that is, IFN-*α*, IL-6, IL-8, and IL-10 [21]. In addition, Biobran enhances phagocytosis of* E. coli* and causes a significant induction of cytokines by neutrophils and monocytes and a reduction of the toxicity of chemotherapeutic agents [22, 23].

Not many studies have examined the effect of immune modulation on IBS symptoms. The aim of this study is to investigate the therapeutic effects of Biobran in IBS patients.

## 2. Methods

### 2.1. Study Design and Patients

This pilot study was a randomized, double-blind, placebo-control trial. Patients aged >20 years who had IBS, as defined by the Rome III criteria for diarrhea predominant IBS (IBS-D) or mixed IBS (IBS-M), were recruited for this study. The patients had recurrent abdominal pain or discomfort associated with loose or watery stools for at least 2 days per week within the preceding 3 weeks. Study patients had to undergo colonoscopy or colonography within 1 year of enrollment to show that there was no organic abnormality to explain the symptoms. Patients who reported the following conditions were excluded: (1) gastrointestinal organic lesions such as peptic ulcer, Crohn's disease, ulcerative colitis, and pancreatitis; (2) history of major abdominal surgery; (3) evidence of cardiovascular, gastrointestinal, metabolic, psychological, or malignant disease; and (4) pregnancy, lactating, or attempting to conceive. Patients who were using medications that could alter gastrointestinal function 2 weeks prior to enrollment were not eligible for this study. Patients taking nonsteroidal anti-inflammatory drugs, steroids, or antibiotics were also excluded, as well as those regarded as unsuitable by the investigators of this study. If concomitant medications had been prescribed for coexisting diseases before obtaining informed consent, they were continued during the study period without changing the dosage and dosage timing. Other concomitant therapies believed to affect the evaluation of this study were prohibited until the end of the study.

Patients were randomly assigned using computerized random numbers between 1 and 40 to receive either 1 g of Biobran powder (3.52 kcal, carbohydrate 752 mg, protein 115 mg, lipid 0 mg, dietary fiber 25 mg, moisture 44 mg, Daiwa Pharmaceutical Co. Ltd., Tokyo, Japan) or placebo twice a day for a 4-week period. This dose of Biobran is a common use for functional food. The placebo powder included dietary fiber and was identical to Biobran in volume, color, and taste. Each IBS symptom was assessed at baseline and weekly intervals following treatment. Gastrointestinal-specific QOL and anxiety were evaluated by a self-reported questionnaire before and at the end of treatment. All aspects of the protocol were approved by the Medical Ethical Committee of the Nagoya City University Graduate School of Medical Sciences (number 211-2). Written informed consent was obtained from all patients prior to the study in accordance with the Declaration of Helsinki.

### 2.2. Symptom Assessment

At the end of treatment, the subjective global therapeutic efficacy was assessed by the patients. The patient's subjective global assessment of the therapeutic efficacy in terms of its condition after treatment was evaluated according to 5 categories: (1) markedly improved, (2) slightly improved, (3) unchanged, (4) not so good, and (5) deteriorated. Categories 1 and 2 were defined as effective; and categories 3, 4, and 5 were defined as not effective. To evaluate the patients' QOL and anxiety state, a gastrointestinal-specific QOL questionnaire, the Gastrointestinal Symptom Rating Scale (GSRS) [24], and a psychological test questionnaire, the State-Trait Anxiety Inventory (STAI) [25], were completed by the patients at baseline and following the 4-week treatment. The GSRS includes 15 items and uses a 7-point Likert scale ranging from “no discomfort” to “very much discomfort.” The 15 items were combined into 5 symptom clusters: reflux, abdominal pain, indigestion, diarrhea, and constipation. A higher score in a GSRS cluster indicates greater discomfort. The STAI questionnaire, consisting of 40 questions, 20 questions for state and 20 for trait anxiety trait, was converted to a scoring system standardized for a Japanese population.

### 2.3. Laboratory Test

A blood sample was collected from all patients before and following 4 weeks of treatment. The complete blood count, blood picture, C-reactive protein (CRP), proportion of B cell to T cell in peripheral blood lymphocytes, and NK cell activity were used to evaluate the changes of inflammation and immunological activity. T cell, B cell percentage in lymphocytes, and NK cell activity were measured by flow cytometry [26] and ^57^Cr-released assay [26], respectively. Plasma catecholamines, adrenalin and noradrenalin, were also examined as stress markers by high performance liquid chromatography (HPLC) [27].

### 2.4. Study End Point and Statistics

The primary end point of this study was the subjective global assessment of the efficacy of Biobran following the 4 weeks of treatment.

The secondary outcomes were change in total and each GRSR abdominal symptom score, change in STAI score, and change in value of each laboratory test.

Values were presented as mean ± SD. The differences in mean values between the Biobran and placebo group were compared by the Student's *t*-test or *U*-test. The IBS symptom scores were assessed with the analysis of covariance. Scores of GSRS and STAI and values of the laboratory test between baseline and following the 4-week treatment were compared using the Wilcoxon ranks test or paired *t*-test, as appropriate. The global assessment categorical variables were evaluated by the chi-squared test. A *P* value < 0.05 was considered statistically significant.

## 3. Results

**Figure 1:**
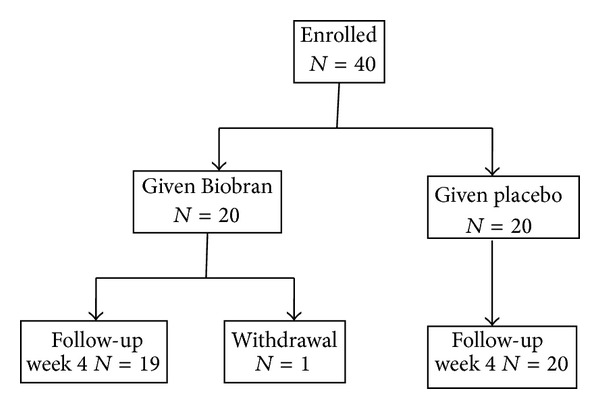
Flow diagram of study subjects.

This study was performed from 2006 to 2007. Forty patients, aged 49.2 ± 15.1 years, were enrolled in this study with randomization of 20 patients each to Biobran and placebo. IBS subtypes according to the Rome III criteria were 28 IBS patients with IBS-D and 12 IBS-M patients.  Table 1 shows the baseline characteristics of the patients (Table 1). There were no significant differences in age, gender, duration of disease, or the number of IBS subtypes between the Biobran and placebo groups. One patient in the Biobran group was excluded from the endpoint analysis, because he did not visit the hospital following the 4-week treatment (Figure 1).

### 3.1. Symptom Assessment and Efficacy of Treatment

The global assessment was effective in 63.2% of the Biobran and 30% of the placebo group (*P* = 0.0465) (Table 2).

Baseline values and changes in GSRS and STAI scores before and after 4 weeks of treatment are shown in Table 3. There were no significant differences in all GSRS scores of both baseline and after 4 weeks of treatment between the Biobran and placebo groups. Significant improvement in the total and category for reflux, diarrhea, and constipation of GSRS scores was observed after Biobran administration. However, no significant changes were observed in total or any of the items in the GSRS scores in the placebo group. In addition, no significant change in the STAI score was observed after Biobran or placebo administration (Table 3).

### 3.2. Laboratory Test

The changes in the values of hematological and serological examinations are shown in Table 4. No significant differences were observed in all baseline values of these data except the platelet count between the Biobran and placebo groups. After the intake of Biobran, the percentage of neutrophil was significantly lower than in placebo group, whereas B-cell percentage in Biobran group was higher than in placebo group. The lymphocyte ratio in peripheral white blood cells (WBCs), B-cell percentage in lymphocytes, and NK cell activity after Biobran intake were significantly increased when compared with the baseline values. In addition, the neutrophil ratio in the WBC and serum CRP values showed a significant decrease in contrast to the baseline value in the Biobran group. These changes were not observed after placebo intake. The placebo group showed a significant decrease in the peripheral blood platelet count. No significant changes were observed in the values of the serum catecholamine concentration in either of the 2 groups.

### 3.3. Adverse Events

There were no adverse effects in either the Biobran or placebo groups.

## 4. Discussion

We have demonstrated the therapeutic effects of anti-inflammatory and immune modulatory treatments by Biobran administration in patients with IBS. This has been manifested by Biobran ability to improve IBS symptoms where subjective assessment of Biobran was effective in more than 60% of patients. In addition, Biobran treated patients showed increase in lymphocyte ratio and NK cell activity. The GSRS scores in both diarrhea and constipation concerning IBS after Biobran intake were significantly improved when compared with the baseline values.

It is widely accepted that low grade inflammation and immunological alterations play important roles in the development of IBS symptoms [13, 14]; IBS is believed to be associated with an activated adaptive immune response. In an inflammatory environment in the gut mucosa, increased epithelial permeability [28, 29] can allow antigens to enter easily and may lead to an increase in various immune cells and abnormal gut flora. These gut dysfunctions and activation of the digestive immune system may affect gastrointestinal motility and visceral sensitivity, which have been proposed as the pathophysiological factors of IBS.

In this study, the results of the laboratory tests revealed the anti-inflammatory and immune modulatory effects of Biobran. After Biobran intake, NK cell activity increased and the CRP value showed a significant decrease when compared with the levels before intake. In addition, significant increase in the ratio of lymphocytes in WBCs and the B-cell percentage in lymphocytes was also observed, as well as a significant decrease in the neutrophil ratio. Ghonum et al. have shown that Biobran is a potent biological response modifier that works through stimulation of different arms of the immune system, such as NK, T, and B cells [18–21]. These previous data on Biobran support our result. A significant decrease in platelet count, however, was observed only in the placebo group. The reason for this effect may be partly due to higher baseline values in the placebo group than in the Biobran group. However, no data are available to explain this result.

A few clinical trials [30–33] have suggested that treatment with various probiotic bacteria can improve IBS symptoms. The intestinal microflora plays an important role in the health of the host [34–36] and possesses an immune modulatory capacity. Probiotic bacteria offer a means of modifying the enteric microflora and their therapeutic effects may influence the immune response [34, 37] by modulating mucosal balance in the intestinal tract. In our study, oral Biobran intake increased the percentage of lymphocyte and enhanced NK cell activity, indicating that Biobran has immune modulatory effects in IBS patients. In addition, Biobran, which is a polysaccharide derived from rice bran, may influence the microflora in the digestive tract. However, the precise biological Biobran functions are not well understood. Further studies are needed to clarify the mechanisms of the beneficial effects of Biobran in IBS patients.

The potential of Biobran to directly mediate psychological stress and the autonomic nervous system was considered low. Psychological factors are important in the pathogenesis of IBS. The concentration of serum catecholamines including noradrenalin rises under psychological stress and the prevailing state [38, 39] of sympathetic nervous activity. In this study, no changes in either the STAI scores or values of serum catecholamine resulting from Biobran intake were observed, suggesting that there is no direct relationship between the effect of Biobran and psychological stress.

The first limitation of this study was that the sample size was small because of pilot study and that there was no data for some of cytokines such as IL in subjects before and after the intake. We could not investigate the correlation between the profile of immune cells and IBS symptom severity.

In conclusion, this is the first study to examine the anti-inflammatory and/or immune modulatory effects in IBS patients. We detected a significant improvement in symptoms in the cases of Biobran treatment when compared with that of the placebo. These data provide a novel application for Biobran in treatment of IBS patients. To confirm our results, further trials should be encouraged in a more generalized population.

## 5. Conclusion

Immune modulatory effects of Biobran, modified arabinoxylan rice bran, are probably useful in improving IBS symptoms.

## Figures and Tables

**Table 1:** Clinical characteristics of subjects.

	Biobran (*n* = 19)	Placebo (*n* = 20)
Age (years)	48.8 ± 14.7	49.6 ± 16.0
Gender (M/F)	9/10	11/9
IBS subtype		
IBS-D	14	13
IBS-M	5	7
Duration of disease (years)	17.8 ± 11.8	15.8 ± 10.1

Values are mean ± SD.

IBS, irritable bowel syndrome.

IBS-D, irritable bowel syndrome with diarrhea.

IBS-M, mixed type irritable bowel syndrome.

**Table 2:** The global assessment to treatment of either Biobran or placebo.

	Biobran (*n* = 19)	Placebo (*n* = 20)
Markedly improved	4 (21.1%)∗	2 (10.0%)
Slightly improved	8 (42.1%)∗	4 (20.0%)

Unchanged	6 (31.6%)	11 (55.0%)
Not so good	1 (5.3%)	2 (10.0%)
Deteriorated		1 (5.0%)

*P = 0.0465 versus placebo.

**Table 3:** Changes in values of Gastrointestinal Symptom Rating Scale (GSRS) and State Trait Anxiety (STAI) between baseline and after 4 weeks of treatment.

	Baseline	Treatment	*P*
GSRS			
Total dimension			
Biobran	3.21 ± 0.93	2.60 ± 0.96	<0.001
Placebo	2.93 ± 0.68	2.77 ± 0.75	N.S.
Reflux			
Biobran	2.33 ± 1.35	1.71 ± 1.17	0.013
Placebo	1.66 ± 0.90	1.55 ± 0.90	N.S.
Abdominal pain			
Biobran	2.33 ± 1.35	1.71 ± 1.17	N.S.
Placebo	1.66 ± 0.90	1.55 ± 0.90	N.S.
Indigestion			
Biobran	3.21 ± 0.93	2.60 ± 0.96	N.S.
Placebo	2.93 ± 0.68	2.77 ± 0.75	N.S.
Diarrhea			
Biobran	4.88 ± 1.98	3.51 ± 2.02	<0.001
Placebo	4.39 ± 1.59	3.95 ± 1.40	N.S.
Constipation			
Biobran	3.87 ± 1.73	3.20 ± 1.67	0.024
Placebo	3.68 ± 1.82	3.28 ± 1.67	N.S.
STAI			
State			
Biobran	3.21 ± 0.93	2.60 ± 0.96	N.S.
Placebo	2.93 ± 0.68	2.77 ± 0.75	N.S.
Trait			
Biobran	3.21 ± 0.93	2.60 ± 0.96	N.S.
Placebo	2.93 ± 0.68	2.77 ± 0.75	N.S.

Values are mean ± SD; No significant changes between Biobran and Placebo.

**Table 4:** Changes in values of hematological and serological examinations between baseline and after 4 weeks of treatment.

	Baseline	Treatment	*P*
White blood cell (×10^2^)			
Biobran	59.9 ± 17.0	58.7 ± 15.8	N.S.
Placebo	63.8 ± 18.3	60.7 ± 14.7	N.S.
Neutrophil (%)			
Biobran	58.1 ± 8.1	54.3 ± 6.8∗	0.039
Placebo	60.5 ± 8.3	60.3 ± 7.9	N.S.
Lymphocyte (%)			
Biobran	32.0 ± 7.4	35.5 ± 6.2∗∗	0.022
Placebo	29.8 ± 7.0	30.3 ± 7.5	N.S.
Hemoglobin (g/dl)			
Biobran	13.6 ± 1.2	13.8 ± 1.3	N.S.
Placebo	14.0 ± 1.9	13.8 ± 2.1	N.S.
Platelet count			
Biobran	19.5 ± 5.7	21.9 ± 4.7	N.S.
Placebo	23.2 ± 5.5	21.4 ± 5.2	0.011
CRP (g/dl)			
Biobran	0.12 ± 0.10	0.10 ± 0.13	0.042
Placebo	0.32 ± 0.47	0.25 ± 0.36	N.S.
NOR			
Biobran	445.8 ± 166.1	508.6 ± 179.5	N.S.
Placebo	412.6 ± 183.0	389.3 ± 140.1	N.S.
T cell (%)			
Biobran	87.9 ± 3.6	86.9 ± 4.7	N.S.
Placebo	87.1 ± 4.6	86.9 ± 3.7	N.S.
B cell (%)			
Biobran	5.28 ± 2.49	6.44 ± 2.75	0.042
Placebo	5.84 ± 2.52	5.28 ± 2.87	N.S.
NK cell activity (%)			
Biobran	31.7 ± 12.5	40.3 ± 15.7	0.002
Placebo	36.2 ± 15.4	35.6 ± 15.7	N.S.
Th1/Th2			
Biobran	9.92 ± 5.60	10.05 ± 5.99	N.S.
Placebo	8.71 ± 5.31	10.24 ± 7.21	N.S.

Values are mean ± SD; *P = 0.0184 versus Placebo; **P = 0.0384 versus Placebo.

CRP, C reactive protein; NOR, Noradrenalin.

## Abbreviations

IBS: Irritable bowel syndrome
QOL: Quality of life
PI-IBS: Postinfectious IBS
NK cell: Natural killer cell
IFN: Interferon
IL: Interleukin
GSRS: Gastrointestinal Symptom Rating Scale
STAI: State-Trait Anxiety Inventory
CRP: C-reactive protein
HPLC: High performance liquid chromatography
IBS-D: IBS with diarrhea
IBS-M: Mixed IBS.
